# Patient-Reported Symptom Changes Following Breast Implant Explantation with Total Capsulectomy: A Prospective Case Series of 50 Patients

**DOI:** 10.3390/healthcare14162603

**Published:** 2026-08-19

**Authors:** Kostadin Gigov, Ivan Ginev, Petra Kavradzhieva, Mariya Miteva

**Affiliations:** 1 Department of Propedeutics of Surgical Diseases, Section of Plastic, Reconstructive and Aesthetic Surgery and Thermal Trauma, Faculty of Medicine, Medical University of Plovdiv, “St. George” University Hospital, “Peshtersko Shosse” Blvd. 66, 4002 Plovdiv, Bulgaria; kavradjieva@gmail.com; 2 Department of Endocrinology, Faculty of Medicine, Medical University of Plovdiv, 4002 Plovdiv, Bulgaria; m_miteva@yahoo.com

**Keywords:** breast implant illness, total capsulectomy, implant explantation, silicone breast implants, case series

## Abstract

**Background:** Breast implant illness (BII) is a constellation of non-specific systemic and local symptoms—including fatigue, joint pain, cognitive dysfunction, and autoimmune-like manifestations—attributed to silicone breast implants. Although BII remains a controversial clinical entity, accumulating evidence suggests symptom improvement following implant removal; however, causal mechanisms and optimal surgical strategies remain uncertain. **Objective**: The objective was to evaluate the prevalence of BII symptoms and the effect of explantation with en bloc total capsulectomy on symptom resolution in affected women. **Methods**: A prospective case series of 50 women who underwent explantation with total capsulectomy between 2023 and 2025 was conducted (Level IV evidence). The BII Questionnaire of the American Society for Aesthetic Plastic Surgery (ASAPS) was administered to assess preoperative and one-year postoperative symptom burden. Perioperative data—including implant characteristics, capsular findings, and imaging results—were analyzed. Symptom changes were assessed using descriptive statistics and nonparametric tests. **Results**: The mean age at explantation was 43 years, with a mean age at implantation of 31 years. Implants were predominantly placed in the retropectoral plane (80%). Intraoperative rupture was identified in 34 patients (68%). At one-year follow-up, a statistically significant reduction in symptom burden was observed, with an 81.7% decrease in mean symptom frequency (preoperative mean: 10.79 vs. postoperative: 1.97; *p* < 0.001). The most pronounced improvements were noted in chronic fatigue, cognitive impairment, and delayed wound healing. No surgical complications were recorded, and aesthetic outcomes were satisfactory among patients who underwent single-stage mastopexy. **Conclusions**: Explantation with en bloc total capsulectomy is associated with substantial symptom improvement in women presenting with BII. Although the precise pathophysiology of BII remains elusive, these findings support a patient-centered approach incorporating thorough preoperative evaluation and shared decision-making. Future research should prioritize the development of standardized diagnostic criteria, biomarker validation, and patient subgroup stratification to optimize clinical management and further advance the understanding of BII. **Limitations:** The absence of a control group precludes causal inference. Objective clinical and immunological assessments were not performed. Patient-reported symptoms were not corroborated by formal clinical evaluation. Most patients were self-referred, introducing referral and expectation bias. The sample size was small.

## 1. Introduction

Breast implant illness (BII) represents a constellation of non-specific systemic and local symptoms attributed to breast implants, particularly silicone-based devices. It affects a subset of implant recipients and is characterized by fatigue, joint pain, cognitive impairment (“brain fog”), memory loss, myalgia, sleep disturbances, and autoimmune-like manifestations. Symptoms typically emerge 5 to 13 years after implantation, though onset may occur at any point following surgery [1,2,3].

BII is distinct from other implant-related complications, such as Breast Implant-Associated Anaplastic Large Cell Lymphoma (BIA-ALCL), and pertains primarily to autoimmune and inflammatory reactions potentially triggered by silicone exposure [3,4]. The condition remains controversial, with ongoing debate regarding its pathophysiology and clinical significance. To date, BII lacks formal diagnostic recognition, and no validated diagnostic criteria exist.

Some authors found no correlation between BII symptoms and silicone breast implants [5].

However, other studies continue to document symptom improvement following explantation [6,7,8,9].

Current research suggests multiple potential mechanisms underlying BII [9,10,11]. The prevailing immunological hypothesis posits that silicone implants act as chronic antigenic stimuli, triggering persistent immune activation [8,12].

Silicone particles embedded in periprosthetic tissue serve as foci of sustained inflammation; immune cells attempting to degrade silicone undergo apoptosis, releasing intracellular silicone that progressively accumulates in surrounding tissues [10].

BII manifests through diverse symptom categories affecting multiple organ systems [3,6,13].

- Musculoskeletal: Joint pain (arthralgia), muscle pain and weakness (myalgia), morning stiffness, and back/neck pain are among the most frequently reported symptoms [8,13].
- Cognitive and Neurological Symptoms: “Brain fog,” memory loss, word-finding difficulties, trouble concentrating, anxiety, depression, and fatigue represent significant components of BII [3,8].
- Systemic Manifestations: Dry eyes and mouth, hair loss, skin problems including rashes, sleep disturbances, and various autoimmune condition symptoms comprise the systemic aspects of BII [3,14,15].

BII lacks standardized diagnostic criteria, making clinical assessment challenging [16]. The absence of validated diagnostic tests necessitates thorough evaluation to exclude alternative diagnoses [3].

This complexity is compounded by the fact that over 100 symptoms have been associated with BII, many of which overlap significantly with other medical conditions [7,17]. A comprehensive clinical evaluation incorporating patient history, symptom patterns, and treatment response is therefore essential. The primary treatment for BII remains surgical removal of implants (explantation), with studies consistently showing symptom improvement in a significant percentage of patients. Recent research indicates that explantation leads to significant symptom relief in approximately two-thirds of patients suspected of having BII [1]. Importantly, controlled studies suggest that symptom improvement may occur independent of whether partial, total, or no capsulectomy is performed. These data highlight the difficulty in attributing symptom improvement to removal of the capsule itself, as opposed to implant removal, patient expectations, or other confounding factors.

It has been reported that 65.5% of patients presenting with three or more distinct preoperative symptoms no longer met this threshold following explantation [18].

Patients who undergo explantation within 10 years of implantation experience better symptom improvement compared to those with longer implant exposure [13,19]. Additionally, patients with comorbidities or high burdens of silicone residues are less likely to experience complete relief after explantation [1].

Complete capsulectomy with en bloc removal of the silicone implants is increasingly recommended as the standard approach for BII patients, as opposed to simple implant removal [20]. This comprehensive approach aims to remove all potentially inflammatory tissue and silicone particles.

Textured implants have been associated with significantly higher odds of complications, revision, and reoperation compared to smooth implants [21]. Higher rates of cellulitis, increased bacterial adherence and biofilm formation, and association with BIA-ALCL represent the primary concerns with textured devices [21,22].

Patients presenting with BII frequently demonstrate elevated rates of anxiety, somatization, and neuroticism, and neuropsychiatric contributions to symptom perception warrant careful consideration. Accordingly, BII should be approached as a diagnosis of exclusion, requiring systematic evaluation to rule out autoimmune, endocrine, neurological, infectious, and psychiatric conditions prior to surgical intervention. The timing of explantation, choice of surgical technique, and postoperative monitoring should be individualized based on symptom severity, patient preferences, and overall health status. The present study aims to contribute to the growing body of evidence on BII—a condition not yet formally recognized—and to explore the potential benefit of en bloc total capsulectomy in achieving symptom resolution.

This case series has been reported in line with the PROCESS guidelines [23] [See Supplementary Materials].

### Aim

The aim was to describe the pattern and frequency of patient-reported symptom changes following breast implant explantation with en bloc total capsulectomy in a consecutive series of women presenting with symptoms attributed to BII.

## 2. Materials and Methods

### 2.1. Study Design

A prospective case series was conducted comprising 50 patients who underwent explantation with en bloc total capsulectomy for silicone breast implants (SBIs) by a single surgeon at a single institution between January 2023 and May 2025.

### 2.2. Inclusion Criteria

Most patients were self-referred and presented with local or systemic complaints they associated with SBIs. Patients were considered for surgery when the following criteria were met:

- Persistent, functionally significant symptoms that patients consistently attribute to their implants and that impact quality of life despite conservative measures.
- Reasonable exclusion of alternative diagnoses that could fully explain the symptom constellation, acknowledging that some comorbidities may coexist.
- Informed understanding by the patient of the current evidence: some studies report substantial symptom improvement after explantation, but causality remains unproven and not all patients experience complete resolution.
- A clear preference for implant removal, whether driven by symptoms, anxiety regarding future health risks, or desire to discontinue implant use.
- We document this shared decision-making process explicitly, including discussion of risks, expected postoperative course, alternative options (e.g., observation or non-surgical management), and realistic outcome scenarios.

Only after comprehensive clinical evaluation was explantation offered (Table 1).

At the initial clinic visit, a detailed patient history was obtained, and a general assessment including physical examination was performed. Medical history—encompassing implantation history and current medications—was gathered through patient interview and review of prior documentation. The surgeon conducted a structured interview with particular attention to implant-related complaints, symptom duration and onset, lifestyle factors (smoking, alcohol, and substance use), and allergies. Physical examination included inspection and palpation of the breast implants, with documentation of the Baker capsular contracture grade. All patients provided written informed consent. This self-referral strategy introduces referral and expectation bias, which is acknowledged as a significant limitation of the study.

**Figure 1:**
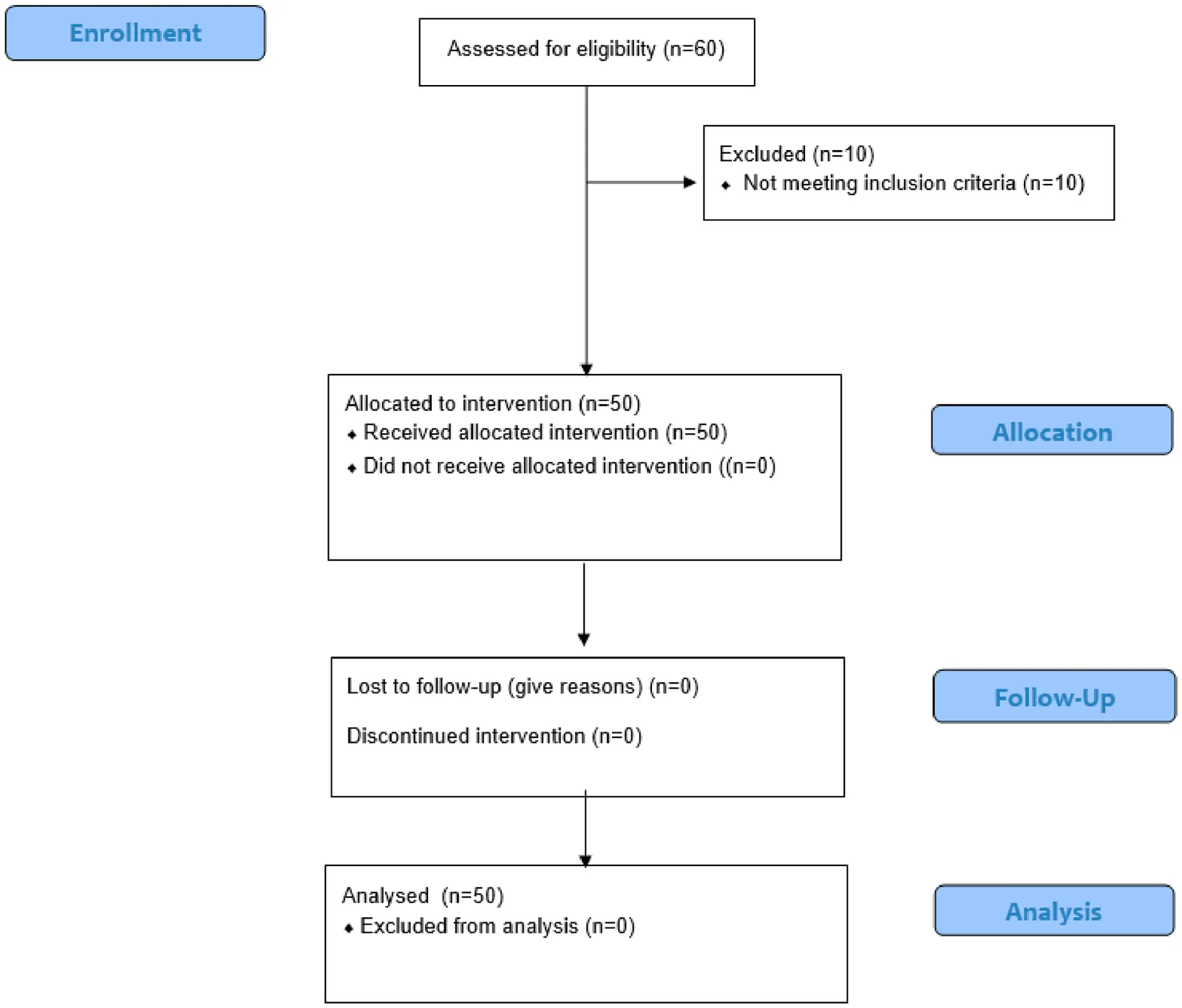
Consort subjects participation figure.

Of all screened patients (n = 60), 10 were excluded due to failure to meet inclusion criteria—autoimmune diagnoses prior to implantation (Figure 1).

### 2.3. Surgical Methods

Patients were counseled regarding explantation and additional surgical options, including mastopexy. The extent of surgery reflected individual patient preference and surgeon judgment rather than an evidence-based determination of necessity for symptom improvement. The study does not compare outcomes between alternative surgical approaches. The decision to proceed with mastopexy was made through a shared decision-making process. If a mastopexy was planned, preoperative planning and marking of inverted-T mastopexy with wise pattern was performed. Patients were marked standing, defining the breast meridian, existing and desired nipple–areola complex (NAC) position, and inframammary fold (IMF). The new NAC is usually set at or slightly above the level of the IMF projection on the breast meridian. A wise keyhole template is used: a periareolar circle (commonly 38–42 mm diameter) with two limbs extending inferiorly to form the vertical component and a horizontal ellipse along the IMF, adjusting limb length (about 6–7 cm areola–IMF) to control vertical scar length.

All procedures were performed under standard sterile technique. Explantation was carried out through an existing or newly created inframammary incision, or through the concurrent mastopexy incision where applicable, to ensure adequate access and visibility. Dissection was performed using monopolar electrocautery, with bipolar forceps employed for hemostasis. Fibrous capsule dissection was performed predominantly with scissors, with the assistant maintaining hemostasis using bipolar forceps throughout. En bloc total capsulectomy was performed in all cases, achieving single-piece intact removal of the fibrous capsule to minimize contamination from implant contents and biofilm, and to ensure complete excision of immunogenic material. Periprosthetic fluid volumes exceeding 50 mL were submitted for cytological analysis. All ruptured implants were managed in accordance with applicable biomaterial disposal regulations. Implant exchange was not performed in any patient. The implant pockets were irrigated with an antibiotic solution consisting of gentamicin, half-strength povidone-iodine (betadine), followed by a final rinse with normal saline.

If a mastopexy was planned, it was carried out in the following fashion: de-epithelialization of the skin within the superior keyhole (between the limbs above the areola) and around the planned NAC, preserving the dermis over the parenchymal pedicle. A superior parenchymal pedicle is then designed, maintaining adequate width and thickness to protect NAC vascularity and innervation. Excess glandular tissue, especially from the lower and lateral pole, is resected as indicated for reduction and shaping; then, the breast is coned by approximating and suturing the medial and lateral pillars to create projection and upper-pole fullness. Skin closure is performed with single interrupted dermal sutures and intradermal uninterrupted sutures for skin approximation.

All patients had Redon drains that were removed when output was under 25 cc/24 h, which, in our experience, occurs 24 to 48 h postoperatively.

Patients were advised to wear compressive bras for 2 months postoperatively and limit physical activity for 6 to 8 weeks.

Numerous perioperative variables were recorded, including implant brand/size, occurrence of rupture (per side), and abnormalities of the implant.

### 2.4. Questionnaire

Prior to surgery, all patients were asked to complete the BII Questionnaire of the American Society for Aesthetic Plastic Surgery (ASAPS) at the clinic. A recognized limitation of this instrument is the lack of psychometric validation and the absence of objective outcome measures. One year postoperatively, the questionnaire was re-administered to assess symptom resolution. Patients were contacted by telephone, and the questionnaire was distributed via an online platform; all patients responded within the designated timeframe with no missing data. The questionnaire encompasses 35 of the most commonly reported BII-associated symptoms.

The response for each symptom is a yes/no option, not a graded one [See Appendix A].

### 2.5. Data Analysis

The data were analyzed using SPSS 24.0. For the evaluation of perioperative data, implant data, and other characteristics, descriptive statistics of the patient cohort are presented.

### 2.6. Patient Demographics

Data including age, BMI, exposition (Table 2), comorbidities (Table 3), smoker and drinker status (Table 4) were evaluated.

## 3. Results

The mean age of implantation was 31 (SD 6.4) years of age. The mean age of explantation was 43 (SD 7.75) years of age. The most common type of implant was Allergan (Table 5). All silicone implants were macrotextured, with the exception being Motiva implants, which were microtextured.

In 40 of the patients, the implants were placed in a retropectoral plane and in 10, in a subglandular plane. Intraoperatively, in 34 patients, a rupture was found. Descriptive statistics were performed to compare symptom occurrence with implant rupture (Table 6), implant position (Table 7), and time of exposition (Table 8). Patients were assigned into one of three groups—exposition under 10 years, 10–15 years, and more than 15 years.

In total, 35 distinct symptoms were analyzed, with the most frequent pre-intervention complaints being “chronic fatigue” (31 patients), “cognitive impairment” (27 patients), and “delayed healing after physical activity” (23 patients).

Post-intervention, chronic fatigue decreased by 83.9%, cognitive impairment reduced by 85.2%, and delayed healing showed a 91.3% reduction (Table 9).

While most of the symptoms improved substantially, some symptoms, like “Hair loss”, had a limited response—52.6% reduction.

A significant improvement was observed in overall symptom relief.

**Figure 2:**
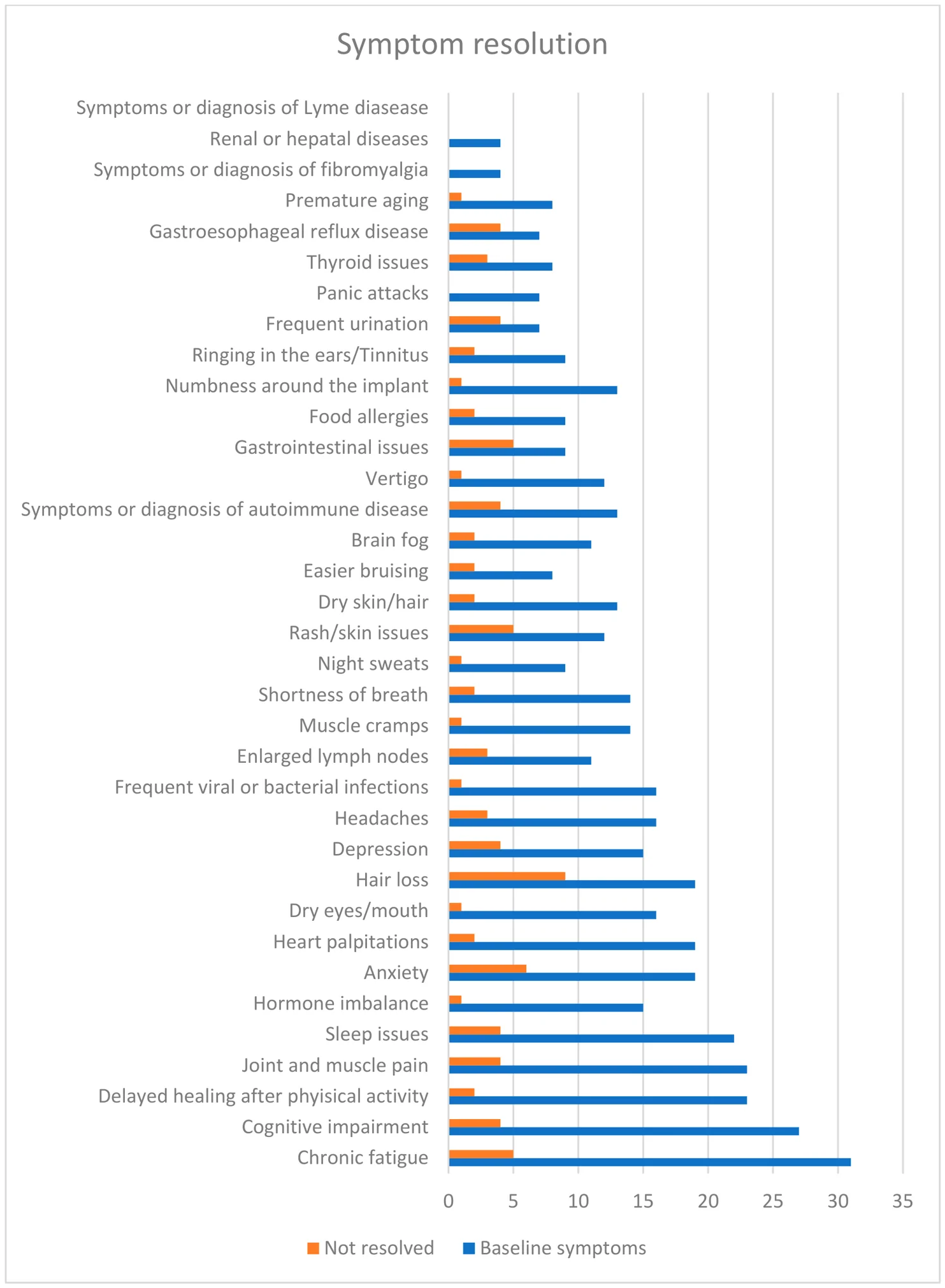
Visual representation of symptom resolution after the intervention.

The mean reduction in symptom count per patient was 8.82 (SD: 4.60), with the mean number of symptoms declining from 10.79 preoperatively to 1.97 postoperatively—an 81.7% reduction in mean symptom frequency (Figure 2). Prior to surgery, all patients reported at least one BII-related symptom. At one-year follow-up, patients demonstrated a markedly reduced likelihood of reporting three or more distinct symptoms compared to the preoperative period. Overall, 84% of patients who had reported three or more distinct symptoms preoperatively no longer met this threshold following explantation. Within this cohort, half of all patients reported immediate symptom resolution. Among the remaining patients, resolution occurred between one month and one year postoperatively: 10 patients reported resolution at one month, eight at six months, and seven at one year.

Capsular contracture was a common intraoperative finding in most of the patients. Aesthetic outcomes were satisfactory in patients who underwent single-stage mastopexy. No surgical complications were recorded, including postoperative infection, seroma, hematoma, wound dehiscence, or NAC compromise. All patients were followed up with for up to one year postoperatively.

Given the absence of a control group, it is not possible to determine whether observed symptom changes were attributable to implant removal, capsulectomy, placebo effects, regression to the mean, or concurrent medical or psychological interventions. Nevertheless, this study contributes to the growing body of observational literature documenting symptom improvement in self-selected patients undergoing breast implant removal.

## 4. Discussion

BII remains one of the most complex and evolving clinical entities in modern plastic and reconstructive surgery, with profound implications for patient care, device regulation, and healthcare provider communication.

In this prospective case series, 50 patients were evaluated before and one year after explantation with en bloc total capsulectomy with respect to symptom dynamics. Symptom resolution was stratified by potential confounding factors, including duration of implant exposure, intraoperative rupture, and implant position. Although the study is purely descriptive in nature, the results demonstrated substantial symptom resolution, with a reduction exceeding 80% in mean symptom frequency.

BII is not currently recognized as a distinct disease entity with formal diagnostic criteria; the associated symptoms are broad, non-specific, and frequently overlap with other medical and psychosocial conditions, limiting the conceptual precision of studies in this field. The present work treats BII as a symptom-based clinical construct—a constellation of patient-reported systemic complaints attributed to breast implants—rather than as a pathophysiologically defined disease entity, focusing on patterns of clinical presentation, referral, and explantation outcomes within this self-selected population. This framing reflects contemporary clinical practice, in which surgeons increasingly encounter patients who self-identify with BII in the absence of objective diagnostic tests.

The recent emergence of proteomic research identifying FGF-19 as a potential biomarker represents a significant scientific advancement that may illuminate the biological basis of symptoms in some patients experiencing BII [1,4].

This apparent paradox—the simultaneous identification of biological markers in symptomatic patients alongside the absence of population-level differences between implant-exposed and unexposed individuals—suggests that BII may represent a heterogeneous spectrum of conditions with multiple etiologies rather than a single, unified disease entity. Some patients may indeed experience immune or metabolic dysregulation triggered by silicone exposure, while others may present with symptoms attributable to somatization, psychosocial factors, or previously undiagnosed medical conditions that are coincidentally attributed to their implants.

Progress in this field requires a more nuanced approach to both BII research and clinical management. Future investigations should move beyond binary causation frameworks—either silicone causes BII or it does not—toward a more sophisticated understanding of patient subpopulations that may respond differently to silicone exposure based on genetic predisposition, immune status, implant characteristics, and psychosocial factors. Multi-omics approaches integrating genomics, proteomics, metabolomics, and microbiomics may reveal distinct biological subtypes of BII with divergent pathophysiological mechanisms and treatment responses [1].

The development of standardized diagnostic criteria is essential to advance both clinical practice and research [13,16]. Current diagnostic approaches lack consensus, limiting the ability to identify true cases of BII-related morbidity versus other medical or psychosocial conditions manifesting in the context of implant presence. Evidence-based diagnostic criteria should incorporate validated symptom assessment tools, specific laboratory and imaging biomarkers when identified, and explicit exclusion criteria for confounding conditions.

Healthcare providers treating women with breast implants should adopt a patient-centered approach that validates reported symptoms while maintaining scientific rigor in differential diagnosis and management [20]. This approach involves comprehensive evaluation to exclude alternative causes, appropriate specialist referral when indicated, and shared decision-making regarding explantation. Patients should be counseled that while symptoms are real and merit investigation, current evidence does not support a universal causal link between implants and systemic symptoms in all cases [24], and symptom improvement after explantation may be multifactorial, potentially including placebo effects [20].

Clinical experience is crucial in the management of BII. We have adopted several pragmatic rules in our diagnostic algorithm:

- Symptom clusters and trajectories are more informative than individual, isolated complaints. Patients with multiple, temporally related symptoms emerging months to years after implantation and progressively worsening are more likely to be considered for surgery than those with single, stable complaints.
- The presence of capsular contracture, documented rupture, or chronic peri-implant inflammation often coexists with BII-attributed symptoms and may act as a reinforcing factor in surgical decision-making, even when causality is uncertain.
- Psychosocial dimensions are systematically explored: we ask about illness beliefs, online information exposure, and prior experiences with medical care, as these may modulate symptom perception and expectations from explantation.

Pitfalls and diagnostic errors to avoid:

- Attributing all symptoms to implants without adequate work-up: non-specific complaints such as fatigue, pain, or cognitive difficulties are common in the general population and can reflect diverse medical or psychosocial causes. Premature labeling as BII may delay appropriate diagnosis and treatment of other conditions.
- Ignoring objective implant pathology: conversely, focusing solely on systemic symptoms without assessing for rupture, capsular contracture, infection, or seroma may overlook surgical problems that warrant treatment regardless of BII considerations.
- Over-promising outcomes: given that causal links between implants and systemic symptoms remain debated and that improvement after explantation is not universal, it is a diagnostic and ethical error to guarantee complete symptom resolution. We instead present outcome data in probabilistic terms and emphasize uncertainty.
- Neglecting mental health evaluation: failing to recognize coexisting anxiety, depression, or somatoform features can lead to unrealistic expectations and postoperative dissatisfaction, even when local surgical results are optimal.
- Binary thinking (“BII or no BII”): rigid categorization ignores the continuum of symptom severity and multifactorial nature of illness. We prefer a graded assessment of likelihood and impact, which better reflects clinical reality and guides individualized care planning.

In patients who have undergone implant-based breast reconstruction, the authors consider the management of suspected breast implant illness to follow the same structured principles as in aesthetic cases but within a more complex clinical and psychosocial context. Initial management should include thorough evaluation of symptom patterns, exclusion of alternative medical and oncologic causes, and detailed discussion of reconstructive priorities and expectations. When a woman with reconstruction self-identifies with BII and reports functionally significant systemic complaints, the decision to explant is made through shared decision-making, weighing potential symptom relief against the aesthetic and body-image consequences of losing the reconstructed breast mound. In many cases, implant removal can be combined with alternative reconstruction (e.g., autologous flaps or delayed fat grafting) to preserve breast shape, which several reports suggest may allow symptom improvement while maintaining oncologic safety and acceptable cosmesis. Regardless of the chosen path, ongoing psychosocial support and close multidisciplinary follow-up are viewed as essential, as anxiety, cancer survivorship issues, and body-image concerns can significantly modulate both symptom perception and satisfaction with the outcome [25,26].

The regulatory landscape must also evolve to support ongoing surveillance and research. Current implant registries provide valuable data on local complications, but enhanced systems capturing standardized systemic symptom data, longitudinal follow-up, and mechanistic investigations are needed [20]. International collaboration through registries such as the PROFILE Registry for BIA-ALCL demonstrates the value of coordinated data collection in rare but serious complications and should serve as a model for BII research infrastructure.

Current limitations in the evidence base:

As in most current BII studies, diagnosis, symptom burden, and postoperative change were evaluated predominantly through patient-reported outcomes and routine clinical examination, without concomitant biomarker profiling, imaging-based inflammatory indices, or standardized autoimmune panels; consequently, the results should be interpreted as describing associations within a self-selected symptomatic population rather than demonstrating a mechanistic link between breast implants and systemic disease. At the same time, this methodological limitation reflects the present state of research, in which no validated objective tests or consensus immunologic work-up for BII exist and where even large reviews and expert position statements conclude that a satisfactory solution to the problem of objective diagnosis has yet to be established. The authors therefore view this study as a contribution to the clinical and patient-reported evidence base while underscoring the need for future, mechanism-driven work incorporating standardized immunologic, toxicologic, and imaging measures to better align research objectives with biologically grounded endpoints. Rigorous prospective designs and trials are required.

### 4.1. Study Limitations

A principal limitation of this study is the inherently subjective nature of the BII diagnostic construct. In the absence of validated, objective diagnostic criteria, case identification relied predominantly on patient-reported systemic symptoms attributed to implants and on patient-initiated requests for explantation. As with other published BII series, our classification reflects a symptom-based, narrative-driven diagnosis rather than a pathophysiologically defined disease entity, introducing potential reporting, perception, and selection biases that may limit the generalizability of these findings. Symptom presence, severity, and postoperative improvement were captured through patient questionnaires and interviews rather than objective biomarkers or standardized clinical indices. This reliance on self-report is susceptible to recall bias, expectation effects (including placebo and nocebo phenomena), and the influence of external information sources such as social media and online patient communities.

Furthermore, no standardized immunological testing or objective biomarker assessment was performed.

Additionally, the predominance of self-referred patients introduces referral and expectation bias, potentially over-representing individuals with more severe or highly medicalized presentations. The relatively small sample size and use of a non-validated questionnaire further limit the external validity of these findings and reduce the precision of symptom change estimates following explantation.

### 4.2. Future Research Directions

Future research should prioritize the development of a standardized clinical definition of BII, incorporating explicit inclusion and exclusion criteria, symptom cluster classification, and severity grading to reduce study heterogeneity. Validated patient-reported outcome instruments encompassing fatigue, cognitive, and neurovegetative domains are needed to enable meaningful cross-study data comparison. Prospective studies incorporating pre-implantation baseline assessments and serial post-implantation evaluations would allow more accurate characterization of BII incidence, time course, and symptom persistence. Validation of objective biomarkers—serologic, imaging-based, or tissue-derived—is essential to distinguish BII from overlapping conditions. Randomized or well-controlled comparative trials are needed to evaluate different explantation strategies with respect to symptom resolution and complication rates. Pre- and postoperative psychological assessment should be integrated into study protocols, particularly in cases with a prominent psychosomatic component. Finally, research should aim to characterize symptom improvement trajectories and identify predictors of non-response or relapse, including the role of psychiatric comorbidities and pre-existing autoimmune disease.

## 5. Conclusions

In this case series, substantial symptom improvement was reported following explantation. However, without a control group, causality cannot be established. The authors recognize that the absence of objective measures and the lack of systematic immunological assessment in this cohort substantially limit the strength of the causal inferences that can be drawn between the study’s objectives and its findings. Findings are hypothesis-generating and require confirmation in controlled studies. Ultimately, BII represents a clinical challenge that demands intellectual humility and scientific rigor from the medical community. The reality that some patients experience significant morbidity associated with their implants must be acknowledged and investigated seriously while avoiding overattribution of causality without sufficient evidence. The ongoing controversy surrounding BII reflects not a failure of scientific inquiry but rather the complexity of teasing apart biological mechanisms, individual susceptibility, and psychosocial influences in a population of women undergoing a cosmetic or reconstructive procedure. Through continued research, development of diagnostic consensus, and evidence-based clinical practice, the field can better serve patients experiencing breast implant-related concerns and advance understanding of the long-term health implications of breast implant surgery. Level of evidence: Level IV.

## Figures and Tables

**Table 1:** Inclusion criteria.

Age	>18
Gender	Female
Clinical factors	Patients with Silicone breast implants who underwent explantation surgery with en bloc capsulectomy
Comorbidities	Patients without clinical diseases that may exert symptoms like those attributed to BII (Confirmed autoimmune diagnoses prior to implantation, Uncontrolled major psychiatric disorders, Active infections, known malignancy, Confirmed or suspected BIA-ALCL)
Implant type	All types of implants
Signed consent	Patients with signed informed consent form
Symptoms	Patients with symptoms that are unexplained and could be attributed to BII

**Table 2:** Patient demographics.

Age	BMI	Implant Exposition Time	Education Level	Ethnicity
42.7 (25–59)	24.2 (22–27)	12.6 (2–28)	45 Bachelor’s degree 5 Upper secondary	Caucasian (n = 50)

**Table 3:** Patient comorbidities.

Psychiatric Diagnosis	Thyroid Disease	Hypertension	Medications	Oher Comorbidities
Not reported	Hashimoto’s disease (n = 4)	grade I (n = 3)	None	Not reported

**Table 4:** Patient smoker (in pack-years) and drinker (in units/week) status.

Smoker Status	Drinker Status
N = 12	N = 9
M = 6.92	M = 11
SD = 2.5	SD = 2.34

N—number; M—mean; SD—standard deviation.

**Table 5:** Implant brand distribution.

	Frequency	Percent	Rupture (n,%)
Mentor	5	10.0	5 (100%)
Allergan	18	36.0	13 (72.2%)
Motiva	3	6.0	0 (0%)
Arion	5	10.0	5 (100%)
Eurosilicone	6	12.0	3 (50%)
Inamed	1	2.0	1 (100%)
Dow Cornig	1	2.0	1 (100%)
Nagor	2	4.0	1 (50%)
PIP	2	4.0	2 (100%)
Natrelle	1	2.0	0 (0%)
McGhan	5	10.0	3 (60%)
Polytech	1	2.0	0 (0%)
Total	50	100.0	34 (68%)

**Table 6:** Distribution of symptom resolution stratified by implant rupture findings.

RESOLVED SYMPTOMS	**Rupture**			**Statistic**	**Std. Error**
	no	Mean		0.7451	0.08030
		95% CI	Lower Bound	0.5749	
			Upper Bound	0.9153	
		Std. Deviation		0.33110	
	yes	Mean		0.7943	0.05170
		95% CI	Lower Bound	0.6890	
			Upper Bound	0.8997	
		Std. Deviation		0.29698	

**Table 7:** Distribution of symptom resolution stratified by implant positioning.

RESOLVED SYMPTOMS	**Position**			**Statistic**	**Std. Error**
	Subglandular	Mean		0.8516	0.07453
		95% CI	Lower Bound	0.6831	
			Upper Bound	1.0202	
		Std. Deviation		0.23567	
	Submuscular	Mean		0.7591	0.05086
		95% CI	Lower Bound	0.6562	
			Upper Bound	0.8620	
		Std. Deviation		0.32167	

**Table 8:** Symptom resolution stratified by exposition time.

Exposition				Statistic	Std. Error
RESOLVED SYMPTOMS	10 years	Mean		0.8206	0.05521
		95% CI	Lower Bound	0.7055	
			Upper Bound	0.9358	
		Std. Deviation		0.25299	
	10–15 years	Mean		0.7903	0.08221
		95% CI	Lower Bound	0.6112	
			Upper Bound	0.9695	
		Std. Deviation		0.29641	
	15+ years	Mean		0.7108	0.09480
		95% CI	Lower Bound	0.5087	
			Upper Bound	0.9129	
		Std. Deviation		0.37920	

**Table 9:** Symptom count and symptom resolution.

Symptom	Resolved N	Preoperative N	*p*	SE	Lower 95	Upper 95
Chronic fatigue	26	31	83.9%	0.066059	70.9%	96.8%
Cognitive impairment	23	27	85.2%	0.068367	71.8%	98.6%
Delayed healing after physical activity	21	23	91.3%	0.058753	79.8%	100.0%
Joint and muscle pain	19	23	82.6%	0.079034	67.1%	98.1%
Sleep issues	18	22	81.8%	0.08223	65.7%	97.9%
Hormone imbalance	14	15	93.3%	0.064406	80.7%	100.0%
Anxiety	13	19	68.4%	0.106639	47.5%	89.3%
Heart palpitations	17	19	89.5%	0.070406	75.7%	100.0%
Dry eyes/mouth	15	16	93.8%	0.060515	81.9%	100.0%
Hair loss	10	19	52.6%	0.114549	30.2%	75.1%
Depression	11	15	73.3%	0.11418	51.0%	95.7%
Headaches	13	16	81.3%	0.097578	62.1%	100.0%
Frequent viral or bacterial infections	15	16	93.8%	0.060515	81.9%	100.0%
Enlarged lymph nodes	8	11	72.7%	0.134282	46.4%	99.0%
Muscle cramps	13	14	92.9%	0.06883	79.4%	100.0%
Shortness of breath	12	14	85.7%	0.093522	67.4%	100.0%
Night sweats	8	9	88.9%	0.104757	68.4%	100.0%
Rash/skin issues	7	12	58.3%	0.142319	30.4%	86.2%
Dry skin/hair	11	13	84.6%	0.100068	65.0%	100.0%
Easier bruising	6	8	75.0%	0.153093	45.0%	100.0%
Brain fog	9	11	81.8%	0.116291	59.0%	100.0%
Symptoms or diagnosis of autoimmune disease	9	13	69.2%	0.128008	44.1%	94.3%
Vertigo	11	12	91.7%	0.079786	76.0%	100.0%
Gastrointestinal issues	4	9	44.4%	0.165635	12.0%	76.9%
Food allergies	7	9	77.8%	0.13858	50.6%	100.0%
Numbness around the implant	12	13	92.3%	0.073905	77.8%	100.0%
Ringing in the ears/Tinnitus	7	9	77.8%	0.13858	50.6%	100.0%
Frequent urination	3	7	42.9%	0.187044	6.2%	79.5%
Panic attacks	7	7	100.0%	0	100.0%	100.0%
Thyroid issues	5	8	62.5%	0.171163	29.0%	96.0%
Gastroesophageal reflux disease	3	7	42.9%	0.187044	6.2%	79.5%
Premature aging	6	8	75.0%	0.153093	45.0%	100.0%
Symptoms or diagnosis of fibromyalgia	4	4	100.0%	0	100.0%	100.0%
Renal or hepatal diseases	4	4	100.0%	0	100.0%	100.0%
Symptoms or diagnosis of Lyme disease	0	0	0.0%	0	0.0%	0.0%

**Figure A1:**
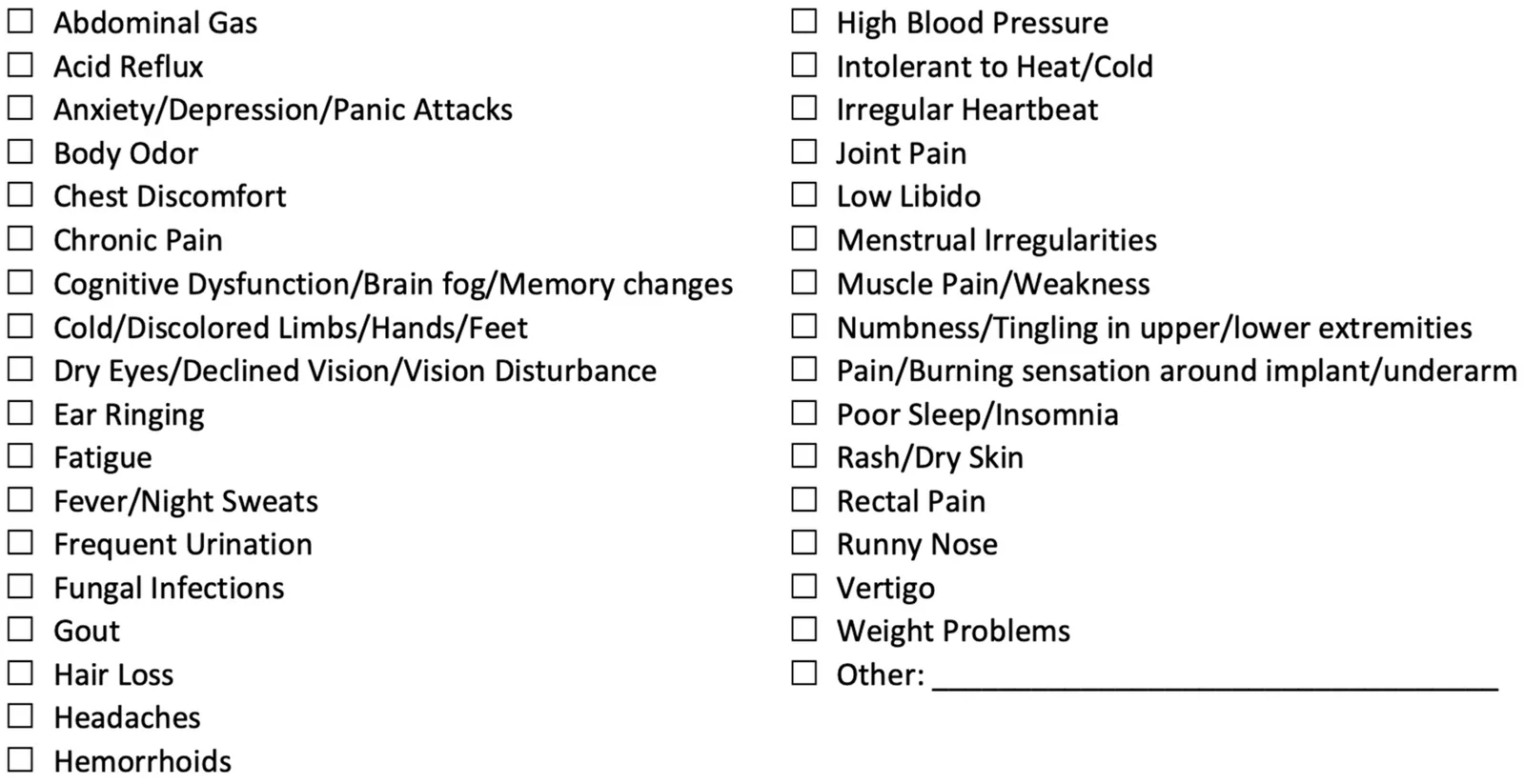
Symptoms questionnaire, part of the breast implant illness questionnaire of the “American society for aesthetic plastic surgery Inc.”.

**Figure A2:**
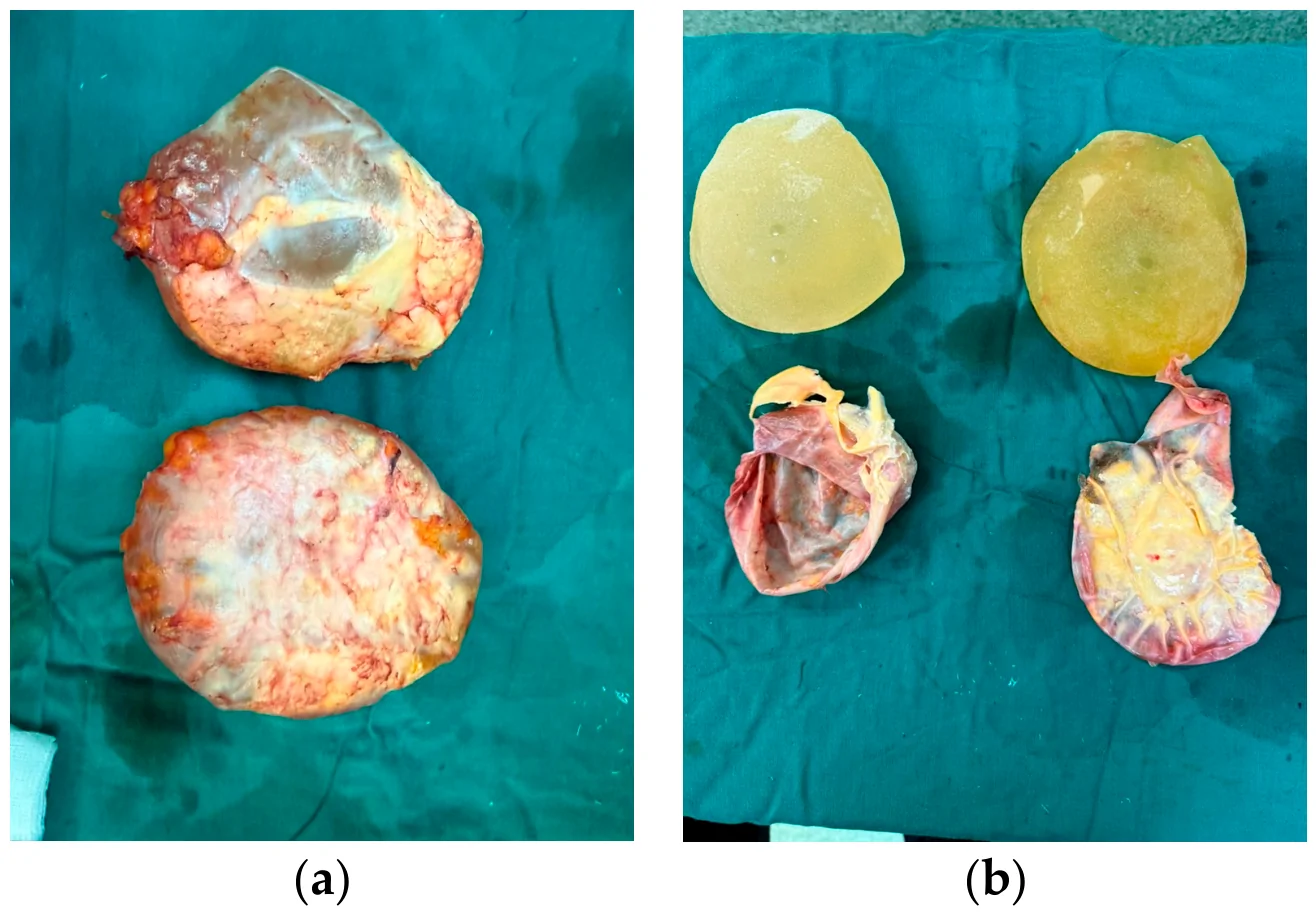
(**a**) Explantation with en bloc capsulectomy. (**b**) Capsular contracture grade IV Baker.

**Figure A3:**
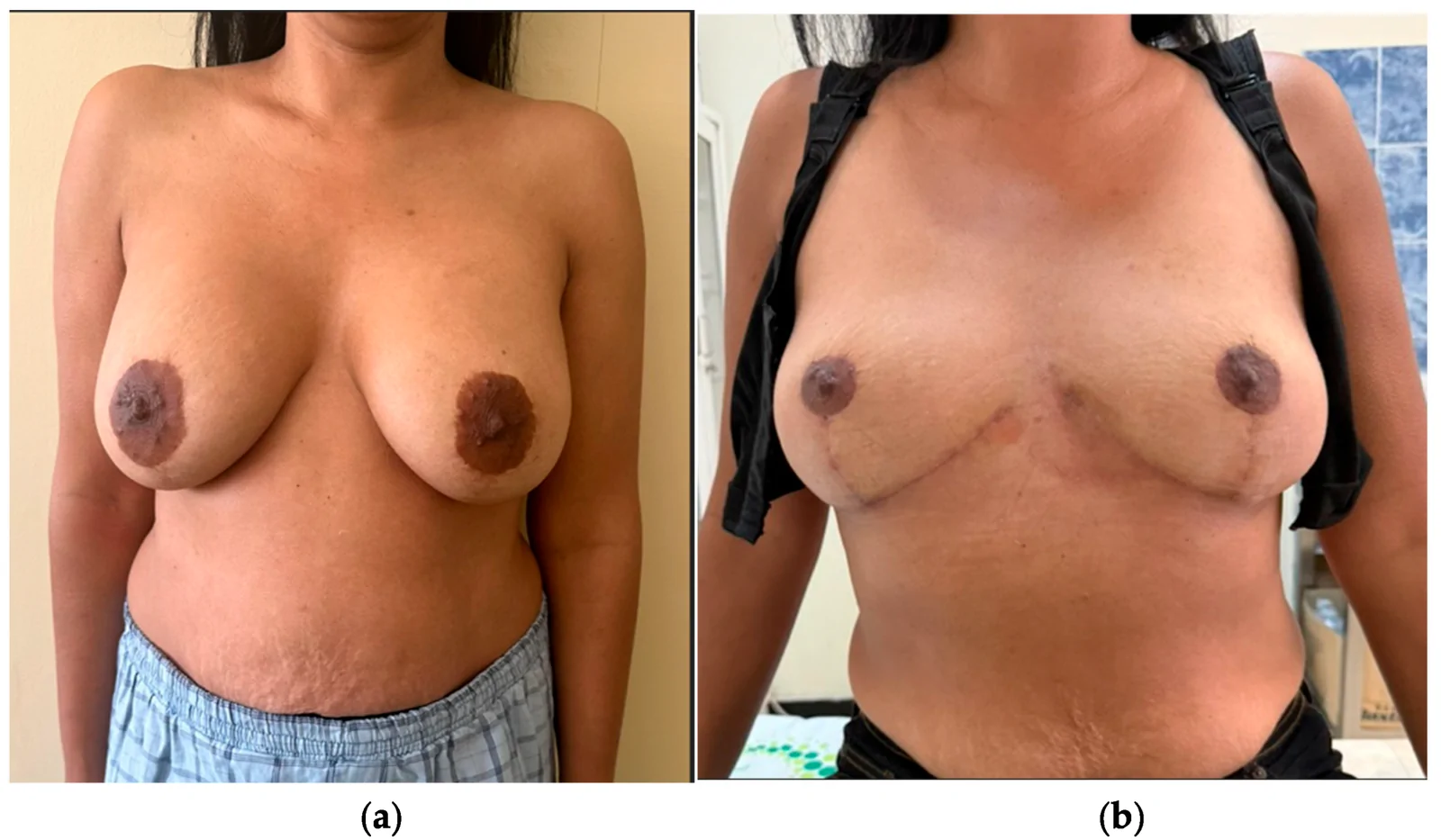
Photos of a patient with en bloc capsulectomy and single-stage mastopexy. (**a**) Preoperative photo. (**b**) Postoperative photo.

**Figure A4:**
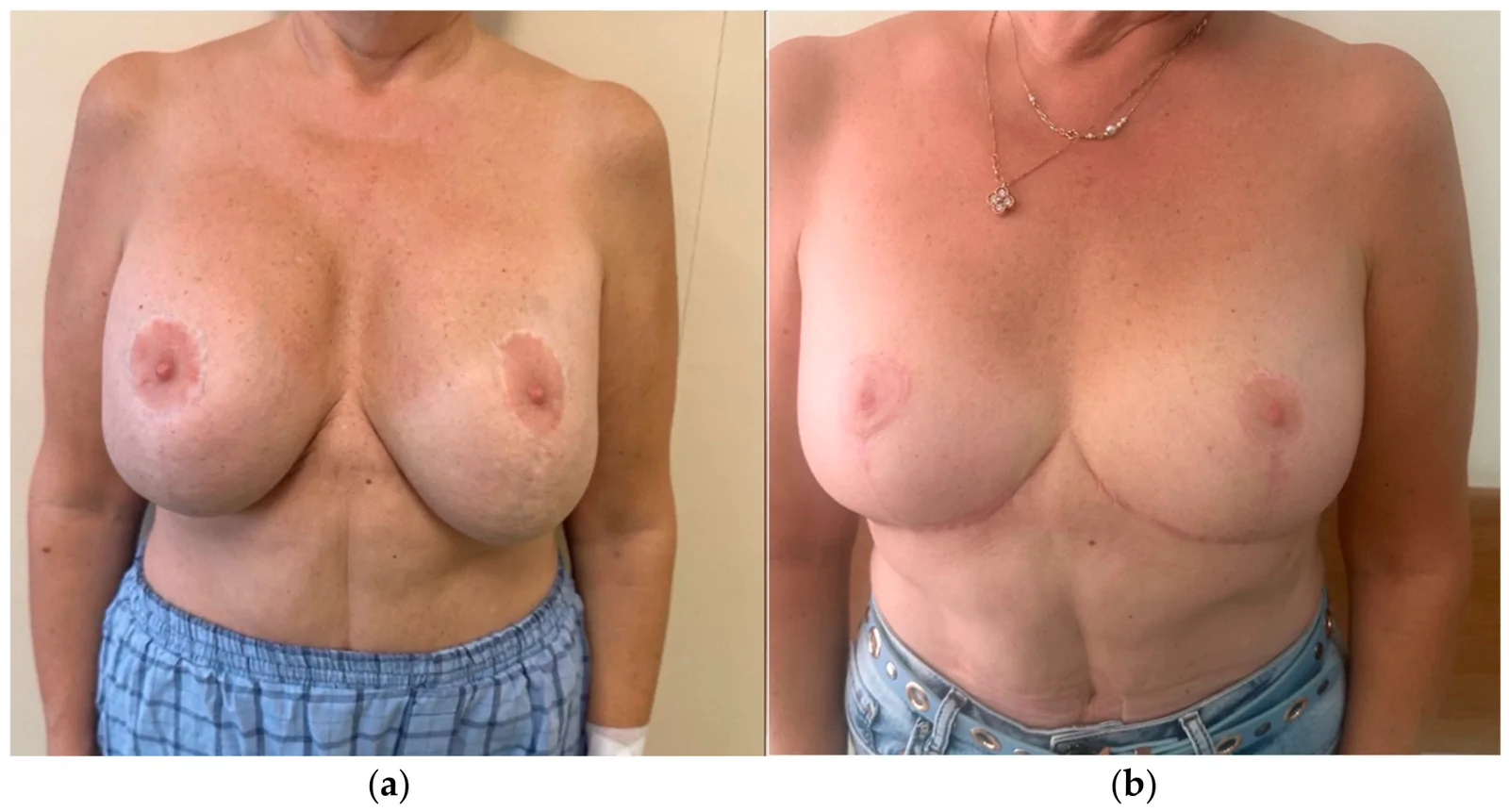
Photos of a patient who had inverted-T mastopexy in the past. (**a**) Before en bloc capsulectomy and single-stage mastopexy; (**b**) 6 months postoperatively.

**Figure A5:**
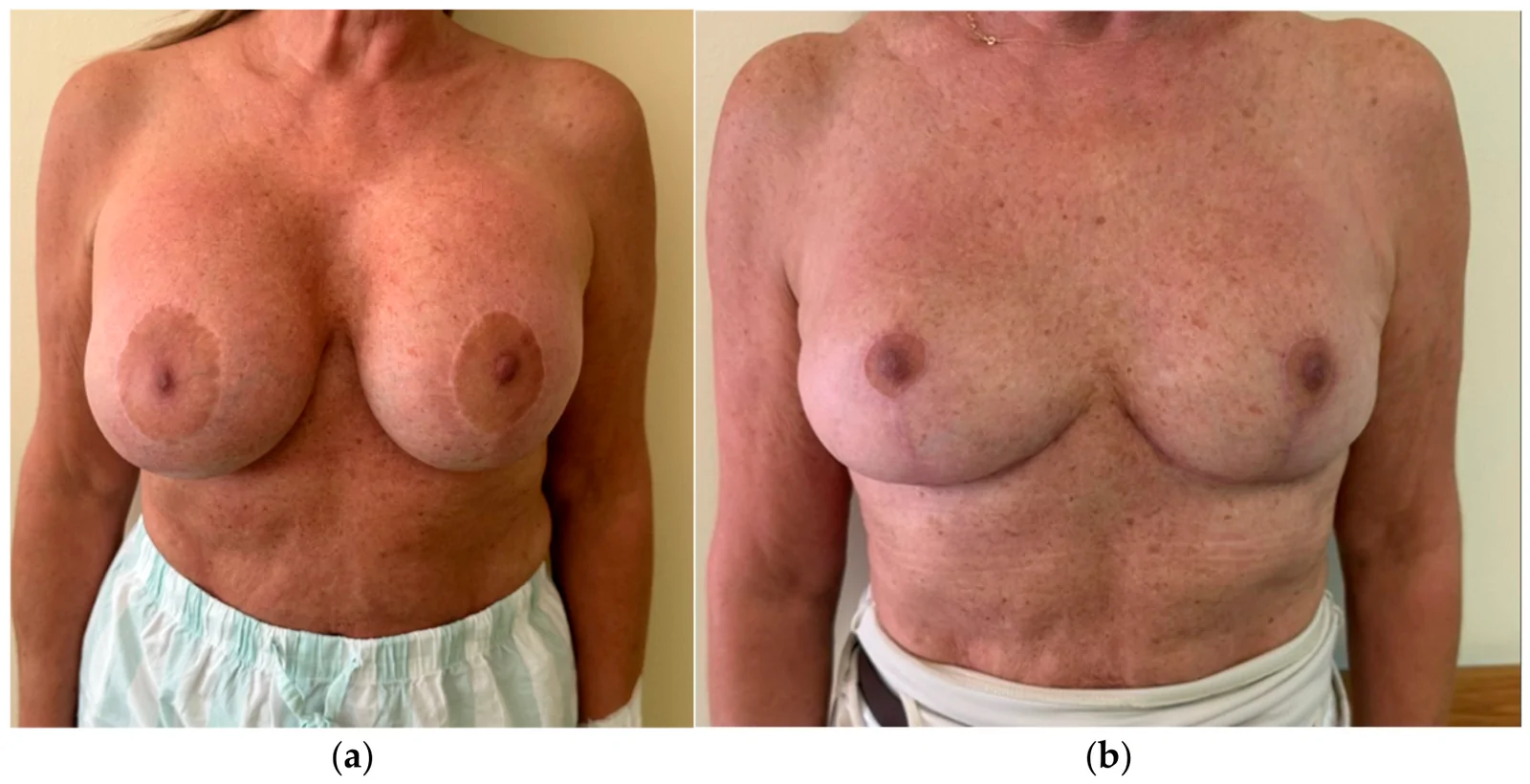
Photos of a patient who had round block mastopexy in the past. (**a**) Before en bloc capsulectomy and single-stage mastopexy; (**b**) 6 months postoperatively.

## Data Availability

The data contains personal information and is not publicly available due to privacy and ethical restrictions, but are available from the corresponding author upon reasonable request.

## Supplementary Materials

The following supporting information can be downloaded at https://www.mdpi.com/article/10.3390/healthcare14162603/s1. PROCESS CHECK LIST.

## Abbreviations

The following abbreviations are used in this manuscript:

BII	Breast implant illness
FGF-19	Fibroblast growth factor-19
ASIA	Autoimmune/Inflammatory Syndrome Induced by Adjuvants
BIA-ALCL	Breast Implant-Associated Anaplastic Large Cell Lymphoma
NAC	Nipple–areola complex
IMF	Inframammary fold
